# Efficacy of auricular acupuncture on sleep quality among individuals with depression: a clinical trial

**DOI:** 10.11606/s1518-8787.2025059006694

**Published:** 2025-10-24

**Authors:** Ana Elise Machado Ribeiro Silotto, Artur Heps, Daniel Maurício Oliveira Rodrigues, Pedro Henrique de Mesquita Pacheco, Nathalia Martins Pereira Sanches, Mariana Cabral Schveitzer, Paulo Rossi Menezes, Alexandre Faisal-Cury

**Affiliations:** I Universidade de São Paulo. Faculdade de Medicina. Departamento de Medicina Preventiva. São Paulo, SP, Brasil; II Universidade do Sul de Santa Catarina. Faculdade de Ciências Biológicas e da Saúde. Departamento de Ciências Biológicas e da Saúde. Palhoça, SC, Brasil; III Universidade Cruzeiro do Sul. Faculdade de Ciências da Saúde. Departamento de Ciências da Saúde. São Paulo, SP, Brasil; IV Universidade Federal de Juiz de Fora. Instituto de Ciências Exatas. Departamento de Estatística. Juiz de Fora, MG, Brasil; V Secretaria de Estado da Saúde de Santa Catarina. Instituto de Psiquiatria de Santa Catarina. São José, SC, Brasil; VI Universidade Federal de São Paulo. Escola Paulista de Medicina. Departamento de Medicina Preventiva. São Paulo, SP, Brasil

**Keywords:** Auricular Acupuncture, Traditional Chinese Medicine, Depression, Sleep Disorders, Randomized Clinical Trial, Mental Health

## Abstract

**OBJECTIVE:**

To evaluate the efficacy of auricular acupuncture in reducing insomnia symptoms among individuals with depression compared with non-specific auricular acupuncture, at four weeks, six weeks, and three months after the intervention began.

**METHODS:**

A randomized, blinded clinical trial assessed sleep quality using the Pittsburgh Sleep Quality Index in 74 adults equally divided into two groups: specific auricular acupuncture and non-specific auricular acupuncture. Both groups underwent 12 auricular acupuncture sessions performed twice a week.

**RESULTS:**

After three months, the intention-to-treat analysis found no statistically significant difference in achieving good sleep quality between groups (33.3% *versus* 26.1%, p > 0.05). However, a trend toward statistical significance occurred after 4 weeks, with 50% of participants in the experimental group and 24.1% in the control group achieving good sleep quality (p = 0.057).

**CONCLUSIONS:**

We observed no statistically significant difference in sleep quality between individuals with depressive symptoms undergoing specific auricular acupuncture compared with non-specific after three months. However, a significant trend (p = 0.057) confirmed by the per-protocol analysis (p = 0.038) indicated that participants receiving specific auricular acupuncture had a 69% higher chance of achieving good sleep quality after four weeks. These findings should be interpreted with caution, as they did not reach statistical significance; rather, they reflect a trend toward significant improvement and are subject to the study's limitations.

## INTRODUCTION

Depression, a prevalent mental disorder characterized by symptoms such as sadness, loss of interest, sleep disturbances, and fatigue, has shown a significant global increase of almost 50% between 1990 and 2017^1^. This disorder often coexists with insomnia, a condition that affects about 72% of the Brazilian population and 30% to 35% of the global population, although only 5% seek primary care^1–3^.

Due to dissatisfaction with symptom remission, side effects, and high costs of depression treatments, the search for "more natural" treatments that offer quality of life and well-being has grown substantially^4–6^. In the last decade, the use of Integrative and Complementary Health Practices has seen considerable growth^7^. Studies indicate that countries like Australia and the United States prefer integrative therapies when treating depression^5,6,8^.

In this context, auricular acupuncture (AA) is a practical therapeutic option that involves inserting fine semi-permanent needles into specific ear points. It offers advantages over body acupuncture, including ease of application, shorter treatment time, lower complexity, relative safety, and continuous physiological stimulation^8^.

The auricular pavilions have a distribution of various nerves, with the concha area having a rich distribution of the vagus nerve. Anatomical studies show that the auricular pavilion is the only location on the surface of the human body with a distribution of the afferent vagus nerve. Thus, needle stimulation in the auricular pavilion seems to lead to specific activation in the brain, primarily through the auricular branch of the vagus nerve^9–11^. Studies indicate that transcutaneous auricular vagus nerve stimulation has shown safety and efficacy in conditions such as epilepsy, major depressive disorders, and insomnia^12,13^.

AA has been associated with reduced insomnia symptoms in various studies^14–17^; however, they have focused on either insomnia or depression, lacking research on its effectiveness for treating insomnia in individuals with depression. Hence, this study assesses the impact of AA intervention on improving sleep quality in people with depression.

## METHODS

This research constitutes a randomized clinical trial (RCT) in which participants, evaluators, data managers, and the statistician were blinded to the treatment allocation. An independent data and safety monitoring board oversaw its progress. The participants were recruited from the community via the university research centers in Santa Catarina, Brazil, from March 1, 2023, to April 28, 2023. The study protocol received ethical approval from the Hospital das Clínicas, Universidade de São Paulo and Universidade do Sul de Santa Catarina. All participants provided written informed consent without any form of incentives or compensation. The study adhered to the reporting guidelines established by the Consolidated Standards of Reporting Trials and the Standards for Reporting Interventions in Clinical Trials of Acupuncture for its design and documentation.

This study is part of a RCT intended to evaluate the effectiveness of AA on depressive symptoms^18^.

### Study Population and Sample Selection

Eligible participants were adults aged 18 to 50 years. The study sample comprised individuals who scored within the range of moderate to moderately severe depression on the PHQ-9 assessment (score PHQ-9 10-19). Exclusion criteria included individuals with prior exposure to AA or other complementary and integrative health practices, those at risk of suicidal ideation, or individuals experiencing severe depression.

### Randomization and Blindness

A computer program executed block randomization with a 1:1 allocation ratio using various block sizes (4, 6, and 8), corresponding to the two study groups: the experimental group or specific auricular acupuncture (SA) for depression and usual care, and the control group or non-specific auricular acupuncture (NSA) and usual care. All participants continued their usual healthcare for ethical reasons. Participant allocation was concealed in sealed envelopes, with no influence from participants or investigators on the randomization, which was managed by an independent statistician. Participants were informed of the two treatment protocols.

### Instruments

#### Sleep Quality Assessment

Sleep disturbance levels was assessed using the Pittsburgh Sleep Quality Index (PSQI) questionnaire, translated into Portuguese without alterations from the original version and validated by Bertolazi et al.^19^

Developed in 1998, the PSQI is arguably the most widely used self-report instrument, proving suitable for evaluating sleep quality. The questionnaire consists of 19 questions regarding sleep quality and disturbances over the past month. The sum of scores can vary from 0 to 21. A total score from 0 to 4 indicates good sleep quality, 5 to 10 indicates poor quality, and a score above 10 points indicates possible sleep disturbance^19^.

#### Other Instruments

The Patient Health Questionnaire (PHQ-9) determined eligibility for the RCT, focusing on assessing depressive symptoms. Comprising nine questions, this questionnaire was developed to evaluate the presence of symptoms associated with major depression, following DSM-IV criteria. Each question investigates the frequency of symptoms over the past two weeks. Additionally, a questionnaire collected demographic, socioeconomic, and health data, including race/ethnicity, age, gender, marital status, income, education, prior diagnoses of depression or mental illnesses, medication use, habits, and weekly working hours.

### Outcomes

#### Main Outcome

The primary outcome concerned the difference in the percentage of participants who achieved a global score below 5, as assessed by the PSQI questionnaire, three months after inclusion. A follow-up score at three months showing a score below 5 (PSQI < 5) was considered successful or symptomatic remission. PSQI < 5 indicates good sleep quality.

#### Secondary Outcomes

- Difference in the percentage of participants with a global score below 5 points (PSQI < 5) after four and six weeks from inclusion between the two groups;
- Differences in sleep quality scores assessed by PSQI after four and six weeks and three months from inclusion between the two groups.

### Procedures

The evaluators underwent training to approach participants uniformly, minimizing recruitment biases. A standardized set of questions and procedures was employed across all stages, including pre-screening, screening, data collection, and protocol application.

Individuals with severe depression (PHQ ≥ 20) or suicidal ideation were excluded from the study and were instead monitored and attended by a psychiatrist. AA sessions took place twice a week, alternating between auricular pavilions, spanning a six-week period, and totaling 12 sessions. Each session lasted 15 minutes and occurred in designated rooms.

Participants were instructed by acupuncturists to manually stimulate each point for 30 seconds or until the auricular pavilion showed signs of redness or sensitivity to pressure, three times a day (morning, afternoon, and evening) every day. The acupuncturists in the study had specific training, with a minimum of 1,200 hours in acupuncture and a minimum of ten years of experience. They underwent standardized training before the study began.

All participants completed PHQ-9 and PSQI questionnaires in the fourth and sixth weeks, as well as three months from the study initiation. Blinding assessment, treatment efficacy perception, and participants’ perceptions about their received treatment were collected three months into the study to evaluate the success of blinding.

### Specific Auricular Acupuncture

In the SA group, we employed a protocol based on traditional Chinese medicine's diagnosis of depression. All participants were treated with six predetermined acupuncture points: Shenmen, Sub Cortex, Heart, Lung, Liver, and Kidney. The EL^11^ device from the NKL brand facilitated precise point location by detecting areas of lower electrical resistance on the skin, indicating more neuroreactive points or authentic acupuncture points^17^. Semi-permanent needles with dimensions of 0.20mm X 2.5mm were inserted to a depth of 2.5mm^20,21^.

### Non-specific Auricular Acupuncture

Recognizing the challenges in establishing control protocols due to the auricular pavilion's high responsiveness and innervation, our study employed a NSA group as a control strategy. The NSA group received superficial needling at "non-points" or "irrelevant true points" to stimulate other neural segments, acknowledging that sham needling is not physiologically inert. Their points included the external ear, cheek/face area, and four non-specific points in the helix region, which are unrelated to mental health symptoms. A locator device confirmed that the selected sham areas were not neuroreactive points. The control group used needles sized 0.20mm X 1.0mm, targeting a more superficial needling depth of 1.0mm^20–22^.

### Ethics Committee Declaration

The study protocol received ethical approval from the Hospital das Clínicas, the Universidade de São Paulo (Opinion N° 6.083.343), and the Universidade do Sul de Santa Catarina (Opinion N° 3.781.279).

### Informed Consent Form

Resolution CNS no. 466/12 and Helsinki recommendations were followed in preparing this form in duplicate. Researchers and participants signed both copies. Participants retained the second copy with the respective protocol number. The ICF also included the contact information of the researchers, CEP-USP, and CEP-UNISUL.

### Statistical Analysis

An independent statistician, who remained blinded to the groups, performed statistical data analysis. Sample size was based on the primary outcome of the original study "Efficacy and safety of auricular acupuncture in depression: a randomized clinical trial." The size was designed to detect a 30% difference (experimental group 60%, control group 30%) in the recovery of depressive symptoms (PHQ-9 < 10) between groups. A minimum sample of 36 participants per group was estimated, considering the test as two-tailed, 80% power, and a significance level of 5%. An additional 10% was added considering dropout losses, totaling 40 in each group, for a total of 80 participants.

Fisher's exact test and Wilcoxon-Mann-Whitney tests were employed for group comparisons at baseline. Intent-to-treat and per-protocol analyses assessed the efficacy of treatments in relation to the remission of poor sleep quality (PSQI < 5) at different timepoints of the study. Friedman's Anova test and post-hoc tests evaluated within-group differences over time, while sensitivity analyses with data imputation assessed the robustness of the results. Various imputation methods were explored, and all analyses were conducted with a significance level of 5% using the R program.

## RESULTS

Of the 304 volunteers initially assessed, 230 were excluded for various reasons, totaling 74 patients included. Of these, 46 composed the per-protocol sample. Eligible participants were randomly allocated to SA and NSA groups (37 in each group). During follow-up, dropouts totaled 13 individuals in the SA group (including one participant with interrupted intervention due to local inflammation) and 15 patients in the NSA group (Figure).

**Figure:**
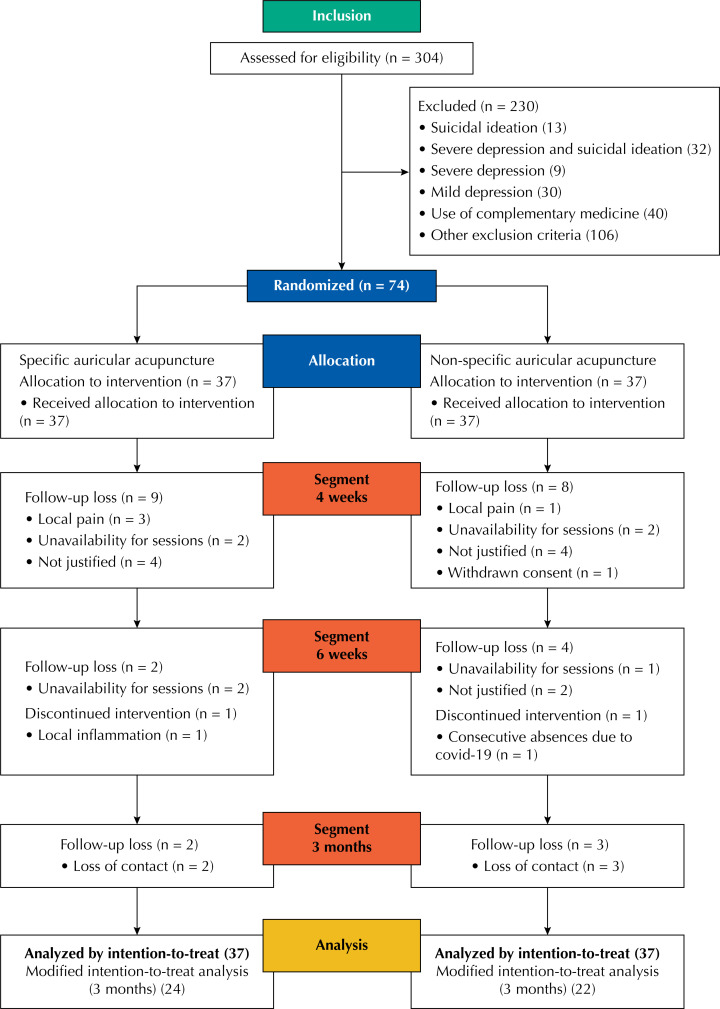
Flowchart of screening, randomization, and follow-up.

The unpaired Wilcoxon-Mann-Whitney test was performed on the evaluation scores of participants who did not complete the study, which showed similar scores among these participants with no statistically significant differences (p = 0.779). Moreover, we observed no statistically significant difference in the dropout rate between the groups during weeks four and six, and at the end of the 3-month follow-up period (p > 0.999 – Fisher's exact test). After four weeks the dropout rate was 24.3% in the SA group and 21.6% in the NSA group; by week six, it decreased to 5.4% in the SA group and 10.8% in the NSA group. After three months, it remained 5.4% in the SA group but dropped to 8.1% in the NSA group. Total dropout rate at the end of the study was 35.1% for the SA group and 40.5% for the NSA group.

The demographic characteristics, clinical diagnosis, pharmacological treatments, and health attributes were comparable between the groups at the beginning of the study, except for race/skin color (Table 1).

**Table 1:** Baseline demographic and clinical characteristics at the time of randomization in the intention-to-treat analysis.

Characteristic		Specific auricular acupuncture (n = 37)	Non-specific auricular acupuncture (n = 37)
Age (years)			
	Median (Q1–Q3)	28 (22%–37%)	30 (23%–36%)
Depression score			
	Median (Q1–Q3)	14.0 (12%–16%)	16.0 (14%–18%)
Gender, n (%)			
	Man	5 (13)	6 (16)
	Woman	32 (87)	30 (81)
	Non-binary	0 (0)	1 (3)
Race/ethnicity, n (%)			
	Black	0 (0)	5 (14)
	Multiethnic	2 (5)	6 (16)
	White	35 (95)	26 (70)
Income^a^, n (%)			
	Up to 3 minimum wages	14 (38)	11 (30)
	More than 3 minimum wages	23 (62)	26 (70)
Marital status, n (%)			
	Single	22 (59)	17 (46)
	Not single	15 (41)	20 (54)
Education, n (%)			
	complete secondary education	10 (27)	10 (27)
	Incomplete higher education	27 (73)	27 (73)
Weekly working hours (n = 49)			
	Median (Q1–Q3)	42 (27%–44%)	44 (22%–44%)
Participant previously diagnosed with depression or other mental illness, n (%)			
	Yes	16 (43)	16 (43)
Participant used any medication for depression, n (%)			
	Yes	8 (22)	4 (11)
Participant used medication in the last 3 months for any of the following purposes n (%)			
	No	21 (57)	27 (73)
	Antidepressant	8 (22)	5 (13)
	Tranquilizer/sedative	5 (13)	4 (11)
	Anxiolytic	7 (19)	3 (8)
Participant consumes alcoholic beverages, n (%)			
	Yes	26 (70)	20 (54)
Participant smokes any cigarette, n (%)			
	Not	35 (95)	35 (96)
	Yes	2 (5)	1 (2)
Former smoker		0 (0)	1 (2)

Q1: first quartile; Q3 third quartile.

a The minimum wage in Brazil is equivalent to US$ 250,47 in 2025.

### Primary and Secondary Outcomes

Three months after inclusion, we observed that 8 (33.3%) of the 37 participants allocated to receive SA and 6 (26.1%) of the 37 participants allocated to receive non-specific auricular acupuncture showed a decrease in the global PSQI score to below 5 (PSQI < 5). This result did not reach statistical significance (p = 0.752). Attainment of good sleep quality (PSQI < 5), as assessed by the PSQI after four weeks, was higher in SA 50%) compared with the NSA group (24.1%). Although the difference did not reach statistical significance, we observed a trend toward significance (p = 0.057) (Table 2).

**Table 2:** Primary and secondary outcomes of global PSQI score reduction to below five in the intent-to-treat analysis.

Outcome		Specific auricular acupuncture (n = 37)	Non-specific auricular acupuncture (n = 37)	Relative risk (95%CI)	p^a^
Reduction of global PSQI score to below five at 3 months compared with baseline. n (%) (n = 47)					
	Yes	8/24 (33.3)	6/23 (26.1)	1.11 (0.76–1.61)	0.752
Reduction in PSQI global score to below five at 4 weeks compared with baseline. n (%) (n = 57)					
	Yes	14/28 (50.0)	7/29 (24.1)	1.52 (0.99–2.32)	0.057
Reduction of global PSQI score to below five at 6 weeks compared with baseline. n (%) (n= 47)					
	Yes		8/25 (32.0)	1.18 (0.77–1.80)	0.565

PSQI: Pittsburgh Sleep Quality Index; 95%CI: 95% confidence interval.

a Fisher exact test.

### Per-Protocol Analysis

The per-protocol analysis confirmed this trend, indicating a statistically significant difference, in which 54.2% of participants in the SA group achieved good sleep quality (PSQI < 5) compared with 22.7% in the NSA group (p = 0.038) within four weeks. Additionally, the relative risk of 1.69 (95%CI: 1.03–2.75) highlights the beneficial effect of the intervention in improving sleep quality. After six weeks and three months, the per-protocol analysis found no statistically significant difference (Table 3).

**Table 3:** Secondary outcome of reduction in PSQI global score to below five in the per-protocol analysis.

Outcome	Specific auricular acupuncture (n = 24)	p^a^	Non-specific auricular acupuncture (n = 22)	p^a^	Relative risk (95%CI)	p^b^
Reduction in global PSQI score to below five after 4 weeks compared with baseline, n (%) (n = 46)						
Yes	13 (54.2)	0.205	5 (22.7)	0.549	1.69 (1.03–2.75)	**0.038** *
Reduction in global PSQI score to below five after 6 weeks compared with baseline, n (%) (n = 46)						
Yes	10 (41.7)		7 (31.8)		1.17 (0.75–1.82)	0.552
Reduction in global PSQI score to below five after 3 months compared with baseline, n (%) (n = 46)						
Yes	8 (33.3)		6 (27.3)		1.09 (0.74–1.60)	0.754

PSQI: Pittsburgh Sleep Quality Index; 95%CI: 95% confidence interval.

a Cochran's Q test.

b Fisher exact test.

* Statistically significant difference (p < 0.05).

The medians of the PSQI scores decreased similarly in both groups without statistically significant differences. However, the median of the SA group was slightly below that of the NSA group in all assessments (Table 4). Nevertheless, our sample size may not have been sufficient in terms of statistical power to identify statistically significant differences.

**Table 4:** Secondary results of global PSQI scores in the intent-to-treat analysis.

Global PSQ scores timeline	Specific auricular acupuncture (n = 37)		Non-specific auricular acupuncture (n = 37)		p-value^a^
Global PSQI score at baseline (n = 74)					
Median (Q1–Q3)	8.0	(6.0–10.0)	8.0	(6.0–11.0)	0.524
Global PSQI score 4 weeks after baseline (n = 57)					
Median (Q1–Q3)	4.5	(4.0–7.0)	6.0	(5.0–9.0)	0.426
Global PSQI score 6 weeks after baseline (n = 51)					
Median (Q1–Q3)	5.0	(4.0 – 5.8)	7.0	(4.0–9.0)	0.177
Global PSQI score 3 months after baseline (n = 47)					
Median (Q1–Q3)	5.0	(3.0–6.3)	6.0	(4.5–7.0)	0.491

Q1: first quartile; Q3: third quartile; PSQI: Pittsburgh Sleep Quality Index.

a Wilcoxon-Mann-Whitney Unpaired.

### Sensitivity Analyses

Sensitivity analyses involved applying four adjustment models for categorical and continuous data. In examining the global PSQI score, we observed notable differences between the groups when performing mean imputation (GEE Model), especially at the six-week assessment where p-value reaches 0.007. At three months, we see a trend toward statistical significance (p = 0.061), suggesting a possible separation between the groups. Importantly, although this difference is inconclusive, it highlights a trend of distinction between the groups. Adopting the Lag-lead model with expectation maximization algorithm indicates positive benefits at the sixth week, evidenced by the p-value of 0.016. However, the sensitivity analysis for categorical data found no statistically significant differences in the four data imputation models (Table 5).

**Table 5:** Sensitivity analysis for PSQI global score with missing data imputation.

PSQI global score Median (Q1–Q3)		Specific auricular acupuncture (n = 37)		Non-specific auricular acupuncture (n = 37)		p-value^a^
Last observation performed						
	Baseline	8.0	(6.0–10.0)	8.0	(6.0–11.0)	0.524
	4 weeks	6.0	(4.0–9.0)	6.0	(5.0–9.0)	0.781
	6 weeks	5.0	(4.0–9.0)	7.0	(4.0–9.0)	0.487
	3 months	6.0	(5.0–9.0)	6.0	(5.0–8.0)	0.695
Generalized estimating equations						
	Baseline	8.0	(6.0–10.0)	8.0	(6.0–11.0)	0.524
	4 weeks	5.5	(4.0–6.0)	6.0	(5.0–7.0)	0.158
	6 weeks	5.0	(4.0–5.8)	6.6	(5.0–7.0)	**0.007** *
	3 months	5.1	(5.0–5.1)	5.8	(5.0–6.0)	0.061
Expectation-maximization lag-lead						
	Baseline	8.0	(6.0–10.0)	8.0	(6.0–11.0)	0.524
	4 weeks	6.0	(4.0–8.0)	6.0	(5.0–9.0)	0.625
	6 weeks	5.0	(4.0–7.0)	7.0	(5.0–11.0)	**0.016** *
	3 months	5.0	(3.0–7.0)	5.0	(4.0–8.0)	0.624
Expectation-maximization lag						
	Baseline	8.0	(6.0–10.0)	8.0	(6.0–11.0)	0.524
	4 weeks	6.0	(4.0–9.0)	6.0	(5.0–9.0)	0.853
	6 weeks	5.0	(4.0–7.0)	6.0	(4.0–9.0)	0.209
	3 months	5.0	(3.0–8.0)	6.0	(5.0–8.0)	0.587

Reduction of global PSQI score to below five n (%)		Specific auricular acupuncture		Non-specific auricular acupuncture		p-value^b^
Last observation performed						
	4 weeks	14	(37.8)	8	(21.6)	0.203
	6 weeks	11	(29.7)	11	(29.7)	> 0.999
	3 months	9	(24.3)	9	(24.3)	> 0.999
Generalized estimating equations						
	4 weeks	14	(37.8)	7	(18.9)	0.121
	6 weeks	11	(29.7)	8	(21.6)	0.595
	3 months	8	(21.6)	6	(16.2)	0.768
Expectation-maximization lag-lead						
	4 weeks	14	(37.8)	8	(21.6)	0.203
	6 weeks	14	(37.8)	8	(21.6)	0.203
	3 months	12	(32.4)	11	(29.7)	> 0.999
Expectation-maximization lag						
	4 weeks	14	(37.8)	9	(24.3)	0.315
	6 weeks	14	(37.8)	11	(29.7)	0.624
	3 months	11	(29.7)	9	(24.3)	0.794

Q1: first quartile; Q3: third quartile; PSQI: Pittsburgh Sleep Quality Index.

a Unpaired Wilcoxon-Mann-Whitney test.

b Fisher's exact test.

* Statistically significant difference (p < 0.05).

## DISCUSSION

Our study included 74 participants undergoing two AA sessions per week, totaling 12 sessions. After three months, a similar proportion of participants in the SA and NSA groups showed a reduction in global PSQI scores below 5, but without reaching statistical significance. Our findings did not show the efficacy of AA in achieving good sleep quality in depressed patients; however, the intent-to-treat analysis found a trend toward benefits after four weeks (p = 0.057), with a relative risk confirming said trend (RR = 1.52; 95%CI: 0.99–2.32). Per-protocol analyses also showed a statistically significant difference, with 54.2% of participants in SA achieving good sleep quality compared with 22.7% in NSA (p = 0.038). However, this difference did not persist in subsequent assessments (six weeks and three months). Despite a trend for lower scores in SA, we observed no statistically significant difference between the groups. Sensitivity analyses suggested a possible trend toward benefits from AA, indicating that evidence of benefit could be confirmed with a larger sample or fewer dropouts.

This project is part of a RCT that showed the efficacy of AA in reducing depressive symptoms by comparing two groups, SA and NSA (45.8% *versus* 13.6%), after three months of participant inclusion^18^. Joint evaluation of the data obtained in the original study and in the present analysis reveals the complexities in managing residual insomnia in individuals with depression. Despite improvements in depressive symptoms, insomnia may persist as a residual symptom. Previous studies indicate that insomnia can be considered a marker of treatment-resistant depression. Acupuncture, while effective in some studies, may have a limited effect when combined with depression, reinforcing the need for multifactorial interventions.

Possible explanations for our results (initial benefit of AA after four weeks, confirmed in the per-protocol analysis, not sustained in subsequent assessments at six weeks and three months) are participant loss and needle discontinuation. The small sample size may have limited the statistical power to identify significant differences between the groups. Moreover, needling can cause some degree of discomfort due to direct contact with the pillow, interfering with participants’ ability to fall asleep or remain in deep, uninterrupted sleep and consequently limiting any therapeutic benefit of AA. Another important aspect is that using sham acupuncture instead of a placebo may have brought some beneficial effect to the control group, since evidence shows that sham acupuncture activates local tissues and the nervous system^23^. Additionally, the widespread acceptance of AA by participants may have contributed to placebo effects.

Our results are consistent with previous research that did not find statistically significant differences in the treatment of insomnia. An RCT involving 150 participants found no statistically significant differences when assessing the efficacy of specific and non-specific acupuncture in treating residual insomnia associated with major depressive disorder^24^. A study with 78 patients diagnosed with major depressive disorder by DSM-IV criteria and complaining of insomnia, undergoing electroacupuncture and superficial needling at NSA points or non-invasive placebo acupuncture, revealed no significant differences between the groups^25^. Another study with 69 participants used auriculotherapy with cowherb seed along with Dexzopiclone 3mg, administered 30 minutes before bedtime, for the experimental group, and sham auriculotherapy combined with dexzopiclone 3mg, also administered before bedtime, for the control group. After eight weeks, there were no statistically significant differences between the groups^26^. An RCT comparing systemic acupuncture, with or without combined AA, to treat insomnia found no statistically significant difference between the group that received acupuncture and the group that received combined treatment. However, both treatments were better than the controlled group, consisting of participants on a waiting list, in reducing insomnia^20^.

On the other hand, there is contradictory evidence showing the efficacy of AA in treating insomnia. A systematic review of 15 RCTs found a positive impact of AA on the treatment of primary insomnia, although the evidence is limited due to the methodological quality of the studies, insufficient sample size, and potential publication bias^14^. A systematic review of 16 RCTs indicated that AA was more effective than placebo, no intervention, pharmacological therapies, and usual therapies in the short-term treatment of insomnia^15^. Additionally, a meta-analysis of 14 RCTs showed that AA was more effective than control groups, especially in cases of insomnia associated with various comorbidities^17^.

Notably, these previous studies did not specifically evaluate individuals with depression. AA efficacy in cases of insomnia related to depression remains uncertain, as most previous research focused on separate treatment scenarios for insomnia or depression conditions. Overall, evidence suggests that AA may stimulate the autonomic nervous system, influence the regulation of the sleep-wake cycle, and promote the release of essential neurotransmitters, and that vagus nerve stimulation plays a relevant role in inducing therapeutic effects^27,28^.

### Strengths and Limitations

Study limitations include the use of self-report instruments (PSQI), susceptible to memory errors. The lack of information collection on factors, such as stress and the use of electronic devices, may limit the comprehensive understanding of the variation in sleep quality scores. The baseline PSQI average (8 points) reflects moderately poor sleep quality, which may limit the magnitude of detectable improvements between the groups. However, the use of PSQI, widely validated and sensitive to changes, allowed us to identify a short-term trend of benefit despite this limitation. Future research should complement the use of PSQI with objective measures such as polysomnography. Additionally, we chose not to incorporate the concepts of "syndromes," an important practice in traditional Chinese medicine that may have negatively impacted the results. Incorporating the treatment of insomnia according to "traditional Chinese medicine syndromes" would add additional complexity to the clinical trial. Another limitation was the impossibility of blinding the acupuncturists. Efforts were made to mitigate the bias associated with non-blinding of AA applicators, including rigorous training and an identical protocol for both groups.

Additionally, the study faced a dropout rate exceeding 30%, which is a significant limitation. Several methods were applied to address missing data and mitigate potential biases, but the high rate of attrition may have impacted the results.

Despite these limitations, the study has strengths like the rigorous implementation of blinding strategies, central randomization, and standardization of the AA protocol. Our study is unprecedented and has an innovative nature in investigating the effects of semi-permanent needle AA on sleep quality in individuals with depressive symptoms. The protocol is easily replicable in different contexts, facilitating the comparison and reproduction of results.

## CONCLUSION

Our results show no statistically significant difference in achieving good sleep quality between SA and NSA after three months; however, after four weeks of intervention the SA group exhibited a trend toward improvement (p = 0.057), which was statistically significant only in the per-protocol analysis (p = 0.038), indicating a 69% higher chance of achieving good sleep quality. Sensitivity analyses reinforce this trend, but these findings should be interpreted with caution as they reflect a trend rather than definitive evidence of effectiveness. The benefits of AA may be more clearly confirmed in a larger sample. Despite the lack of robust statistical significance, our results are promising regarding AA effectiveness, supporting the need for further research to better understand its effects and address the study limitations.

## Data Availability

The datasets generated or analyzed during the present study are not publicly available due to privacy restrictions, but are available from the corresponding author upon request.
